# Tyrosine Phosphorylation in the C-Terminal Nuclear Localization and Retention Signal (C-NLS) of the EWS Protein

**DOI:** 10.1155/2011/218483

**Published:** 2011-05-02

**Authors:** Ruzanna P. Leemann-Zakaryan, Steffen Pahlich, Doris Grossenbacher, Heinz Gehring

**Affiliations:** ^1^Department of Biochemistry, University of Zurich, Winterthurerstraße 190, 8057 Zurich, Switzerland; ^2^Division of Experimental Pathology, Institute of Pathology, CHUV, Faculty of Biology and Medicine, University of Lausanne, Rue du Bugnon 25, 1005 Lausanne, Switzerland

## Abstract

Ewing sarcoma (EWS) proto-oncoprotein, an RNA-binding protein, is involved in DNA recombination and repair, gene expression, RNA processing and transport, as well as cell signalling. Chimeric EWS oncoproteins generated by chromosomal translocations between *EWSR1* and the genes of transcription factors cause malignant tumors. To understand the loss of function by these translocations, the role of the intact EWS protein has to be investigated. The predominantly nuclear localization of the EWS protein via a transportin-1-mediated mechanism is dependent on the recently identified C-NLS (also known as PY-NLS). Among other residues in the C-NLS, Y656 interacts with transportin-1 and is essential for its nuclear localization. Here, we show that Y656 is phosphorylated, which seems to be a critical factor for transportin-1-mediated nuclear import. If Y656 was mutated cytosolic aggregates of the EWS protein, colocalized with transportin-1, were observed, similar to those described with mutants of the closely related FUS/TLS protein that had amino acid substitutions in the PY-NLS causing familial amyothrophic lateral sclerosis.

## 1. Introduction

**Figure 1:**
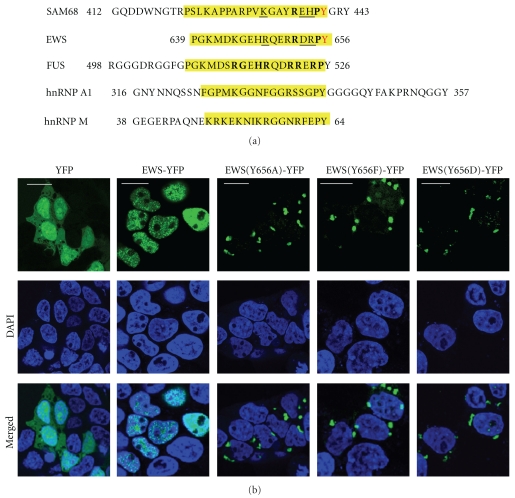
(a) Sequence alignment of PY-NLSs. The homologous regions of C-NLS of the EWS protein, NLS of Sam68 and FUS/TLS protein and M9 NLS of hnRNP A1 and hnRNP M, classified as PY-NLS are in yellow boxes. Phosphorylated Y656 of the EWS protein and Y440 of Sam68 are indicated (in red). Positions of identical residues in SAM68 and EWS C-NLS are indicated in bold and residues with identical charges are underlined. Known positions of the FUS/TLS mutations in ALS are in bold. (b) Subcellular localization of YFP, the C-terminally tagged EWS-YFP, EWS(Y656A)-YFP, EWS(Y656F)-YFP, and EWS(Y656D)-YFP (in green). Nuclei are shown by DAPI staining (in blue). Bars, 15 *μ*m.

The EWS protein is mainly located in the nucleus, accumulated in Cajal bodies and central regions of nucleoli, but it is also present in cytoplasm and associated with cell membrane [1, 2]. We have identified and characterized a nuclear localization and retention signal at the C-terminus of the EWS protein (C-NLS) (Figure 1(a)), which assures nuclear accumulation of the protein [3]. The EWS protein has been shown to be a ligand of transportin-1, a mediator in nucleocytoplasmic protein transport, among many others, including related RNA-binding proteins such as FUS/TLS, hnRNP A1, hnRNP M, and Sam68 [4–6]. The C-NLS of the EWS protein has been classified as PY-NLS, a consensus sequence recognized by transportin-1 [5]. R648, R652, P655 and Y656 have been found to be essential residues in the C-NLS for the nuclear transport of the EWS protein [3]. 

Brk (breast tumour kinases) phosphorylate tyrosine residues present in the NLS of Sam68 [7], which is highly homologous to that of the EWS protein (Figure 1(a)). Y440 of Sam68 corresponds to Y656 of the EWS protein. The residues P and R at position -1 and -4 (from Y) correspond completely, and both proteins have positive charges at position -2 (H/R) and -8 (K/R) as well as a negative charge at -3 (E/D). Phosphorylation and dephosphorylation regulate subcellular localization of numerous proteins [7]. In the present study, we investigated and found that Y656 in the EWS protein occurs in a phosphorylated state and if phosphorylation is abolished, it accumulates in the cytosol colocalized with transportin-1.

## 2. Results and Discussion

Expression of the EWS-YFP fusion protein resulted exclusively in nuclear accumulation, with high concentration in nucleoplasmic speckles and a fraction in the subnuclear central region (Figure 1(b)), thereby interacting with particular proteins such as the RNA helicases p72 and 68 [8]. YFP alone is diffusively distributed between the nucleic and cytoplasmic compartment. Single amino acid substitutions of the C-terminal Y656 by alanine, phenylalanine, and aspartic acid revealed a drastic redistribution of the EWS protein with cytoplasmic accumulations in the perinuclear region (Figure 1(b)). The resulting cytoplasmic aggregation pattern demonstrates that none of these amino acids could successfully substitute the tyrosine residue. Phenylalanine substitution does not fulfill the function of the Y656, implying the importance of the hydroxyl group of tyrosine. Thus, a possible phosphorylation of this amino acid residue in nuclear import function seems likely. However, not even an aspartic acid, which, due to its negative charge, is often used as phosphomimetic of phosphorylated residues, could restore the nuclear localization of the protein. 

**Figure 2:**
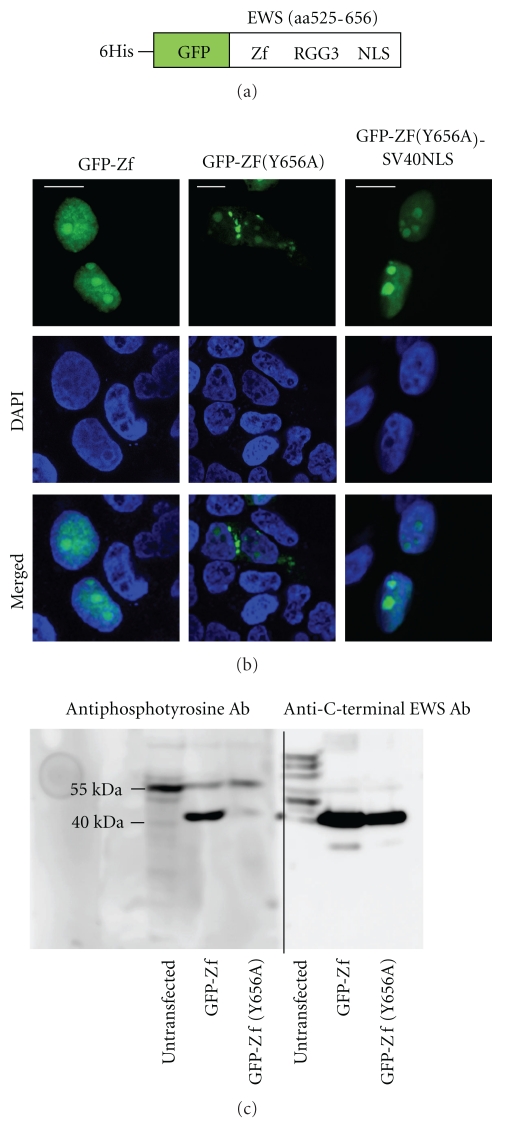
(a) Schematic representation of the N-terminally tagged GFP-Zf fusion protein. 6His-GFP is fused to the EWS mutant protein (aa 525–656). (b) Representative examples of the subcellular localization (all cells expressing the construct are showing the indicated localization) of GFP-Zf, GFP-Zf(Y656A), and GFP-Zf(Y656A)-SV40NLS (in green). Nuclei are shown by DAPI staining (in blue). Bars, 15 *μ*m. (c) Phosphorylation analysis of GFP-Zf and GFP-Zf(Y656A) using antiphosphotyrosine antibodies. GFP-Zf and GFP-Zf(Y656A) fusion protein bands are detectable at ~40 kDa. The additional phosphorylated protein band detected at ~55 kDa was identified as p54nrb.

To demonstrate a possible phosphorylation of Y656, GFP-Zf protein was constructed by fusion of His-GFP with a part of EWS protein (aa 525–656). This part of the C-terminal RNA-binding domain of the EWS protein consists of the Zinc finger (Zf) motif followed by the arginine-glycine rich box 3 (RGG3) and C-NLS (Figure 2(a)). This fragment of the EWS protein (aa 525–656), hereafter called Zf, contains Y656 as the only tyrosine residue and at the same time is large enough to avoid diffusive nuclear import of the GFP-Zf fusion protein. Additionally, the construct GFP-Zf(Y656A), having alanine as the single amino acid substitution for Y656, was produced as a negative control. GFP-Zf shows the subcellular localization pattern of the full-length EWS protein. However, its subnuclear partition is different from that of the full-length protein, as previously described [3] (Figure 2(b)). GFP-Zf(Y656A) reveals the characteristic cytoplasmic distribution as is typical for Y656 mutations of the full-length EWS protein (Figure 2(b)). To show that the cytoplasmic accumulations of the GFP-Zf(Y656A) are not aggregation and precipitation of an insoluble mutant protein, but the result of specific inactivation of NLS function, GFP-Zf(Y656A) was fused with the canonical SV40 NLS. GFP-Zf(Y656A)-SV40NLS shows complete nuclear localization similar to the GFP-Zf without any detectable cytoplasmic aggregates (Figure 2(b)). 

GFP-Zf and GFP-Zf(Y656A) fusion proteins containing 6His-tag at the N-terminus of GFP were expressed in eukaryotic HEK 293(T) cells, extracted, subjected to SDS-PAGE, and analyzed on Western blots by using antiphosphotyrosine antibody. Phosphorylation was detected in GFP-Zf (band at ~40 kDa) but not in GFP-Zf(Y656A) and not in the untransfected sample (Figure 2(c)). The Western blots with the same samples but with anti-C-terminal EWS antibody, that recognized both fusion proteins, demonstrate similar expression level of both proteins (Figure 2(c)). The multiple protein bands detected by anti-C-terminal EWS antibody in the lysate of untransfected HEK 293(T) cells reflect different degradation fragments of endogenous EWS protein (unpublished observations). Phosphorylation of a protein at 55 kDa was also detected. This protein was identified by mass spectrometry as the nuclear RNA- and DNA-binding protein p54nrb. Whatever reasons might be responsible for binding to the resin (possibly endogenous histidine residues located in close proximity), p54nrb can serve here as an internal standard for equal loading.

**Figure 3:**
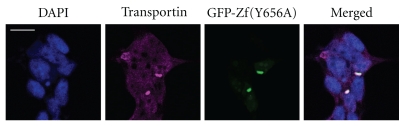
Colocalization of transportin-1 (in magenta) and GFP-Zf(Y656A) (in green). Nuclei are shown by DAPI staining (in blue). Bars, 15 *μ*m. Representative examples of the subcellular localization are shown (all cells expressing the constructs are showing the indicated localization).

As nuclear import of the EWS protein is mediated by transportin-1 [5], we have visualized the subcellular localization of transportin-1. In cells expressing GFP-Zf(Y656A), transportin-1 colocalizes with the mutant GFP-Zf(Y656A) in cytoplasmic accumulations, apart from its characteristic homogeneous nucleocytoplasmic distribution (Figure 3). Conceivably, the part of transportin-1 bound to GFP-Zf(Y656A) is spatially restricted to these cytoplasmic structures and cannot fulfill its functions in nucleocytoplasmic transport due to the missing phosphorylated tyrosine. This finding indicates that phosphorylation is not required for transportin-1 binding which is in accordance with previous data showing that unphosphorylated PY-NLS of EWS or the mutated peptides containing Y656A still bind recombinant transportin-1 [5]. Why are these mutations then causing loss of nuclear import function? Possibly, binding in the absence of a phosphorylated C-NLS cannot induce a conformational change in transportin-1, which might be essential for recruiting or binding to other partners in the nuclear import process and leads, thus, to cytoplasmic accumulations of the transportin-1-EWS complex.

Recently, mutations in FUS/TLS have been shown to be responsible for familial amyotrophic lateral sclerosis (ALS), and mutants of the FUS/TLS protein accumulate in the cytoplasm of cortical neurons and lower motor neurons in the brain of ALS patients [9, 10]. These results indicate that mutant FUS/TLS has a tendency to be insoluble compared to the wild type. Remarkably, mutations are found in the C-NLS (PY-NLS) of FUS/TLS (Figure 1(a)), and the cytoplasmic accumulation is similar with that of the EWS mutant GFP-Zf(Y656A). Our present data indicate that these cytoplasmic aggregations (or, alternatively, accumulations of protein complexes) of FUS/TLS are formed, as in case of the EWS mutants, due to a disturbed nuclear import leading to an increased cytoplasmic concentrations of these proteins. In the normal steady state, the predominant amount of these proteins is transported into the nucleus. Knowledge about the mechanisms of nuclear import, including the role of tyrosine phosphorylation for the function of C-NLS, might have an impact particularly in better understanding the pathogenesis of ALS in order to be able to develop a strategy for its treatment. In addition, it is of interest to confirm the presence of transportin-1 in these cytoplasmic aggregations and to further test the functionality and the role of this restricted protein in progression of the disease.

It is possible that different kinases are able to phosphorylate the EWS protein individually in a cell cycle-dependent and a cell compartment-dependent manner, as it has been found with Sam68 containing PY-NLS. Sam68 is phosphorylated in the nucleus by Brk (breast tumour kinases) and at the cell membrane by Src kinases [7]. Remarkably, kinases of the Src-subfamily are localized in the cytoplasm and can be bound, due to N-terminal myristoylation, to the inner face of the plasma membrane [11], where the EWS protein has also been found [2, 12]. The members of the Brk family, related to the Src family, lack a myristoylation site, and cytoplasmic and nuclear localization is more typical for these kinases [13, 14]. Tyrosine kinases, known to interact with the EWS protein, are Pyk2 and Bruton tyrosine kinase, as well as Lck, a member of Src kinases [15, 16]. Src kinases and Pyk2 recognize a similar tyrosine phosphorylation motif, which suggests that the latter might phosphorylate Y656 of the EWS protein. Conceivably, tyrosine phosphorylation might regulate, apart from its role in nucleocytoplasmic transport, interactions with other proteins or with RNA, as was observed with the related Sam68 and QKI [7, 13, 17].

Although phosphorylation of Y656 in the EWS protein seems to be essential, it might not be the exclusive regulating factor for nuclear localization, since other known or predicted mechanisms can be recruited to cooperatively or sequentially control nucleocytoplasmic distribution of a particular protein. SUMO-ylation, another reversible posttranslational modification of Sam68 [18] and of hnRNP M, has been described to play a role in nucleocytoplasmic shuttling of mRNA-binding protein complexes [19]. Remarkably, the SUMO-ylation motif GKMD is predicted with high probability also in the C-NLS region of the EWS protein (http://us.expasy.org/, SUMOplot) and is a potential subject for further investigation.

## 3. Materials and Methods

### 3.1. Expression Constructs

The eukaryotic vectors for expression of the EWS-YFP, EWS(Y656A)-YFP, and EWS(Y656F)-YFP fusions were constructed as described [3]. To produce the EWS(Y656D)-YFP mutant, the Y656D reverse primer was used. The vectors for expression of His-GFP-Zf and His-GFP-Zf(Y656A) fusions were constructed as follows. For construction of pcDNA3.1(−)B-Zf and pcDNA3.1(−)B-Zf(Y656A) expression vectors, Zf and Zf(Y656A) fragments were amplified by traditional PCR using *EWSR1* cDNA as template and Zf-XhoI-NotI-forward and EcoRI-stop-reverse or Y656A-stop-reverse primers 5′-TCTCTCGAGCGGCCGCGCCACCATGAATCCGGGTTGTGGAAACCAGAA-3′ and 5′-CCGAATTCTCAGTAGGGCCGATCTCTGCGCTCCTG-3′, or 5′-CCGAATTCTCAGGCGGGCCGATCTCTGCGCTCCTG-3′, respectively. The PCR products were treated with restriction enzymes XhoI and EcoRI and subcloned into pcDNA3.1(−)B/myc-His (Invitrogen) to generate in-frame fusions. Finally, the His-GFP-Zf and His-GFP-Zf(Y656A) vectors were constructed by amplification of the His-GFP fragment from EWS-Myc-6xHis/pEGFP-N2, a derivative of pEGFP-N2 plasmid (Clontech) using the NheI-His-forward and NotI-GFP-reverse primers 5′-TCTGCTAGCGCCACCATGGCCGTCGACCATCATCATCATCATCAT-3′ and 5′-TGCGTCGCGGCCGCTCTTGTACAGCTCGTCCATGCCGAG-3′, respectively, and digestion with the appropriate restriction enzymes, and by cloning the resulting product into the pcDNA3.1(−)B-Zf and pcDNA3.1(−)B-Zf (Y656A) plasmids. To obtain His-GFP-Zf(Y656A)-SV40NLS, His-GFP-Zf(Y656A) vector was fused with an NLS from SV40 large T antigen (PKKKRKV). The oligonucleotides 5′-AATTCCCAAAAAAGAAGAGAAAGGTCAGG-3′ and 5′-AGCTTCTGACCTTTCTCTTCTTTTTTGGG-3′ were annealed, and the resulting fragment was digested with restriction enzymes EcoRI-HindIII and was inserted into His-GFP-Zf(Y656A).

### 3.2. Cell Culture and Transfections

Human embryonic kidney (HEK) 293 (T) cells, kindly provided by Professor P. Sonderegger (Department of Biochemistry, University of Zurich, Switzerland), were grown in Dulbecco's Modified Eagle's Medium (DMEM) (Sigma) supplemented with 10% fetal bovine serum (FBS) (Life technologies) and 1% (w/v) each of penicillin and streptomycin (Life technologies) in a humidified 10% CO_2_ atmosphere at 37°C. For visualization analysis, HEK 293 T cells were cultured on glass cover slips to 40% confluency and transiently transfected with mammalian expression constructs using the method of calcium phosphate precipitation. For protein visualization, the cells were fixed 24 h after transfection with 4% paraformaldehyde (PFA) for 5 min and stained with 4′,6-diamidino-2-phenylindol (DAPI) (Roche). Cover slips were mounted using Vectashiled medium (Vector) onto glass slides, and the cells were analyzed by fluorescence and confocal microscopy.

### 3.3. Antibodies, Protein Purification, and Western Blotting

Primary rabbit anti-C-terminal EWS antibody SE 680, kindly provided by Dr. O. Delattre (Institut Curie, Pathologie Moléculaire des Cancers, Paris Cedex), was used with 1 : 5000 dilution. Primary mouse anti-transportin-1 antibody (TNPO1, ab10303, Abcam) was kindly provided by Professor I. Stamenkovic (Department of Experimental Pathology, Institute of Pathology, CHUV, Lausanne, Switzerland). Primary rabbit antiphosphotyrosine antibody (Zymed) was used with 1 : 2000 dilution. The results were confirmed by using primary mouse antiphosphotyrosine antibody (P-Tyr-100, Cell Signaling) with 1 : 2000 dilution (data not shown). The secondary goat antirabbit and goat antimouse antibodies, respectively, coupled to horseradish peroxidase (Sigma) were used with 1 : 2000 dilution. His-GFP-Zf and His-GFP-Zf(Y656A) protein purification by His-SELECT Nickel affinty gel (Sigma) was performed as described [2]. In all the purification buffers, sodium orthovanadate was added as protein phosphatase inhibitor. 

Western blotting was performed as described [12] with some modifications. In case of phosphotyrosine detection, 3% BSA instead of nonfat milk powder was used as blocking agent and in antibody solutions.

### 3.4. Confocal Microscopy

Laser-scanning confocal fluorescence microscopy was performed using a Leica SP2 AOBS UV CLSM microscope and HCX PL APO lbd.BL 63.0x NA 1.40 OIL UV objective. Images were acquired using excitation wavelengths of 405 nm and 514 nm and the emission wavelengths of 470 nm and 528 nm for DAPI and YFP, respectively. Images were captured digitally using Leica software and processed using Adobe Photoshop 8.0. The stacks of images were imported into Imaris (Bitplane) software for the 3D rendering of the images.
